# M. Weinstein and M. A. Lane, *Sociality, Hierarchy, Health: Comparative Biodemography: A Collection of Papers*

**DOI:** 10.1093/emph/eow004

**Published:** 2016-02-28

**Authors:** Jacob Moorad

**Affiliations:** Institute of Evolutionary Biology University of Edinburgh Edinburgh, UK

Interactions with family, friends, caregivers and dependants remind us that we live deeply embedded in networks of complex social interactions, and it seems clear that the quality of our health and the timing of our deaths should be influenced by others. It would also seem obvious as scientists that understanding the mechanisms by which social environments affect us should provide insights into how we might set about lessening suffering and increasing survival. The focus of this edited volume is to demonstrate how recent advances in biodemography, the integration of demographic and biological theory and methods, offer an effective and evolution-based framework for moving this study forward. The volume is motivated by the success of the first edited volume to explore the biodemography of longevity, *Between Zeus and the Salmon* [1], which has been influential but is now nearly 20 years old.

The contributed papers derive from a workshop held in 2014 to expand the field of biodemography ‘to include cross-species comparisons of social environments, social behaviours, and their effects on health, longevity and life histories’ [2]. While a broad range of animal species are represented in these chapters (humans, nonhuman primates, social insects, reptiles and amphibians), an emphasis of the comparative work is clearly placed upon human and nonhuman primates. Far greater diversity is seen amongst the perspectives represented by the collection of contributing authors, which is made of prominent anthropologists, biologists, demographers and gerontologists. The papers cover a wide variety of topics that can be loosely connected into a few themes: intergenerational transfers of resources that contribute to wellbeing; links between social hierarchies, social connectedness and health; and the genetic foundations of longevity. Most review entire fields of study, such as Jenny Tung’s exceptionally detailed coverage of the functional genomics of social environments. Others serve to review and highlight the authors’ own contributions to their fields. New empirical findings are also presented, such as Miller *et al..*’s fascinating presentation of senescence in ectotherms. Evolution is the common perspective adopted here, and discussions within these contributions are clearly linked to evolutionary theory.

While many of the contributions are very worthwhile on their own, the whole does not benefit from much synergy amongst its parts. The chapters are integrated well enough to avoid unwarranted repetition of important background, but little more (it does indeed read as ‘A Collection of Papers’). A related issue is the omission of any clear organizational strategy reflected in the layout of chapters, and the collection has a disordered feel as a result. The introductory chapter by Weinstein *et al..* attempts to define some common themes, but the volume might have benefited had it been broken up into sections by theme, with each proceeded by a brief overview that contextualized the specific contributions.

Another shortcoming is that important approaches to evolutionary demography are neglected, such as quantitative genetic studies of aging in natural population. This is unfortunate as this kind of work has been useful for understanding the nature of polygenic inheritance of aging-related traits in the environmental contexts most relevant to evolution [3]. Indirect genetic effects (IGEs) are particular relevant to the themes addressed in this book as they encompass the heritable components of the social environment [4, 5], but with the exception of a brief mention in Lee’s chapter on intergeneration transfers, they are neglected. A great many of the evolutionary models relating to sociality, health and aging that are discussed in this book may be rigorously investigated by applying quantitative genetic approach to measuring IGEs.

The editors have set out to, ‘revisit both the theoretical underpinnings of biodemography and the empirical findings that have emerged over the past two decades’. They have for the most part succeeded, and this book represents a valuable tool for biodemographers. Most of the ideas presented here are represented in the primary literature. However, because many different perspectives on the social inputs to biodemography are showcased within this volume, readers may benefit from an increased appreciation of the intellectual diversity of the field. For those interested in understanding how evolutionary insights can help us improve health and longevity, it can be recommended as a place to become familiar with current concepts and findings of researchers operating at the juncture between biology and the social sciences. Readers with an interest in evolutionary biology will be satisfied to see that most discussion within these chapters is motivated by evolutionary theory, but it should be noted that this book does not serve as an introduction to the evolutionary theory of aging. While Wachter’s chapter briefly describes the development of this theory, this is done to motivate his models that attempt to relax certain assumptions of the basic theory. Those who are interested in gaining a basic understanding of the theory will be better served by reading the aforementioned volume, *Between Zeus and the Salmon* [1].
